# Correlation of FE_NO_ With Spirometric Measurements and Blood Eosinophil Level in Patients With Severe Asthma

**DOI:** 10.1111/crj.70094

**Published:** 2025-07-24

**Authors:** Wang Chun Kwok, Mary Sau Man Ip, Terence Chi Chun Tam, James Chung Man Ho, David Chi Leung Lam, Kwan Ling Julie Wang

**Affiliations:** ^1^ Department of Medicine, Queen Mary Hospital The University of Hong Kong Pokfulam Hong Kong Special Administrative Region China

**Keywords:** asthma, eosinophil, FE_NO_, FEV_1_, spirometry

## Abstract

**Background:**

Fractional exhaled nitric oxide (FE_NO_) serves as a marker of eosinophil‐mediated airway inflammation and has been used in asthma diagnosis, phenotyping, and guidance regarding selection and adjustment of asthma therapy. Studies suggested that FE_NO_ correlated with asthma symptoms, peripheral blood eosinophil level, blood IgE level, and spirometry indicators of airway obstruction. However, the results are inconsistent across studies.

**Methods:**

A prospective cross‐sectional study was conducted in Queen Mary Hospital among adult patients with severe asthma. Patients had spirometry with bronchodilator reversibility and same‐day FE_NO_. Asthma control test (ACT) score and blood eosinophil and total IgE levels were measured within 4 weeks of FE_NO_ and spirometry. The primary outcome was the correlation of FE_NO_ and spirometric values. The secondary outcomes included the correlation of FE_NO_ with ACT score, blood eosinophil, and total IgE levels.

**Results:**

One hundred thirty‐five severe asthma patients with FE_NO_ performed were included in the study. FE_NO_ was negatively correlated with pre‐bronchodilator FEV_1_ (L) (*r* = −0.188, *p* = 0.029), pre‐bronchodilator FEV_1_ (% predicted) (*r* = −0.169, *p* = 0.050), pre‐bronchodilator FEV1/FVC ratio (*r* = −0.269, *p* = 0.002), and post‐bronchodilator FEV_1_/FVC (*r* = −0.215, *p* = 0.018). FE_NO_ was positively correlated with bronchodilator reversibility (mL) (*r* = 0.248, *p* = 0.006) and bronchodilator reversibility (%) (*r* = 0.823, *p* = 0.002)*,* baseline blood eosinophil level by absolute cell count (*r* = 0.308, *p* < 0.001) and by percentage (*r* = 0.361, *p* < 0.001).

**Conclusion:**

In adult patients with severe asthma, FE_NO_ might have a negative correlation with the FEV_1_, FEV_1_/FVC ratio, and a positive correlation with bronchodilator reversibility, as well as with blood eosinophil levels.

## Graphical abstract

FE_NO_ was negatively correlated with pre‐bronchodilator FEV_1_ (L and % predicted) pre‐ and post‐bronchodilator FEV_1_/FVC ratio, and positively correlated with bronchodilator reversibility (mL and %), as well as baseline blood eosinophil level by absolute cell count and percentage.

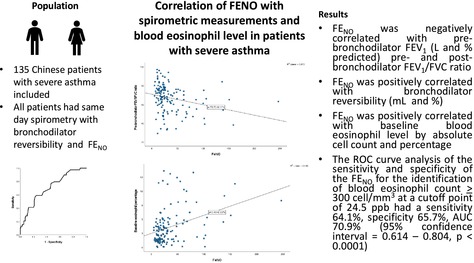

## 1 Introduction

While the gold standard of measuring Type 2 inflammation in the airway is by sputum or bronchial tissue eosinophilia, these tests are not readily available and need expertise to perform and interpret. An observational study comparing surrogate markers for sputum eosinophilia found blood eosinophils and fractional exhaled nitric oxide (FE_NO_) to have comparable diagnostic accuracy, which was superior to total serum IgE in adult asthma patients [1]. Literature reported that endogenous nitric oxide was produced in response to eosinophilic airway inflammation and it can be measured by exhaled breath as in FE_NO_ value measurement [2, 3]. As such, FE_NO_ has gained its popularity as a simple, non‐invasive, and repeatable test to assess and monitor the airway eosinophilia in asthma patients.

Factors affecting FE_NO_ value include age, sex, atopy, and cigarette smoking [4]. An American study suggested that a significant increase in FE_NO_ is noted between groups with respective age ranges of (5, 17) and (17, 25) years, with a breakpoint at 13.97 years. A significant decrease of FE_NO_ is noted between groups with respective age ranges of (45, 55) and (55, 65) years, with a breakpoint at 63.66 years [5, 6, 7]. Smoking has been suggested to decrease FE_NO_ level [8]. Currently, FE_NO_ can be used to predict the response to inhaled corticosteroid (ICS) in asthma, as well as monitor adherence to ICS [3].

While FE_NO_ is considered to be a relatively simple and non‐invasive test to detect eosinophilic inflammation in the airway, controversies do exist regarding the role in managing asthma, especially in correlation with various clinical, spirometric, and inflammatory markers [9, 10, 11, 12, 13, 14, 15]. In Global Initiative for Asthma (GINA) recommendations, FE_NO_ was suggested to be used in asthma phenotyping but not for diagnosis [16]. In the latest update in GINA recommendations, the main FE_NO_ was to guide the treatment decisions in severe asthma. Whereas in European Respiratory Society (ERS) Guidelines, FE_NO_ was also suggested to be a diagnostic tool for asthma [17].

The conflicting results on FE_NO_ and its correlation with various clinical, blood inflammatory, and spirometric markers could be related to the study population and the timing of measurement of these values [18]. The FE_NO_ level would be expected to be different among patients from different age groups [6]. The treatment the patients received could also affect its level [19, 20]. The relationship between blood eosinophil and FE_NO_ levels can vary among patients of different severity and phenotypes [21, 22]. Having a proper timing of assessment (clinical, spirometric, and inflammatory markers) at clinical stable‐state away from exacerbation to evaluate the clinical values of these markers is important.

While FE_NO_ is considered to be useful in asthma management, there are still controversies regarding its role and also correlation with various clinical, blood inflammatory, and spirometric parameters. In this study, we aim to assess the correlation of FE_NO_ values with clinical, blood inflammatory, and spirometric markers among patients with severe asthma at stable state.

## 2 Materials and Methods

This was a cross‐sectional study conducted in Queen Mary Hospital (QMH), Hong Kong. Adult patients with severe asthma managed in the asthma clinic in QMH in 2021 were included. Severe asthma is defined as per International ERS/ATS guidelines on the definition, evaluation, and treatment of severe asthma definition [23].

The inclusion criteria were adult patients at or above 18 years old with a diagnosis of asthma, confirmed by symptoms typical of asthma with demonstration of variable expiratory airflow limitation by spirometry with bronchodilator reversibility testing, preferably by spirometry and exclusion of alternative diagnoses. Patients who fulfilled the ERS/ATS definition of severe asthma for at least 1 year prior to the screening visit were recruited. The included patients all had severe asthma and had been treated with maintenance treatment as per GINA recommendation for severe asthma for at least 6 months before recruitment. Patients who had a predominant diagnosis of chronic respiratory conditions other than asthma that may dominate the clinical picture of respiratory status, pregnant patients, patients with recent respiratory tract infections in the past 90 days, patients with poor asthma medication compliance or poor inhaler technique, as well as patients receiving biological antibodies were excluded from the study.

At the screening visit, patients would have a detailed assessment including history on asthma and co‐morbidities, body mass index measurement, ACT questionnaire, and blood taking for blood eosinophil and total IgE levels. Patients were recruited at clinically stable state without exacerbation in the past 6 months and also no change in asthma treatment in the past 6 months. The patients were also assessed by a respiratory specialty nurse to be compliant with asthma medication. Patients would then undergo spirometry with bronchodilator reversibility test and same‐day FE_NO_ measurement, within 4 weeks of the screening visit. Spirometry was performed with CareFusion Vmax Encore 22 system according to American Thoracic Society (ATS) standard [24], both before and after administration of 400 μg of Salbutamol by inhalation via spacer. FE_NO_ was measured according to the ATS recommendations using NIOX VERO [25].

The primary outcome was the correlation of FE_NO_ and spirometry values. Secondary outcomes included the correlation of FE_NO_ with asthma control test (ACT) score, blood eosinophil, and total IgE level.

Subgroup analysis was performed among patients with T2‐high asthma, which is defined as having a baseline blood eosinophil count ≥ 150 cells/mm^3^ ± IgE ≥ 100 IU/mL [7].

The study was approved by the Institutional Review Board of the University of Hong Kong and Hospital Authority Hong Kong West Cluster (UW 21‐278).

### 2.1 Statistical Analysis

Numeric data based on demographic features and biomarker results were presented as mean, standard deviation following most of these were normally distributed variables. Unpaired *t*‐tests or Wilcoxon Mann–Whitney U‐tests were applied for continuous variables with data in normal or not in normal distribution, respectively. The categorical variables like sex and stratified variables were presented in frequencies and percentages. The relationships between FE_NO_, spirometry values, ACT scores, blood eosinophil levels, and total IgE levels with demographic variables and other biomarkers were assessed using the Pearson's correlation coefficient metrics. Statistical significance was determined at the level of *p* < 0.05. The correlation between the parameters expressed as categorical variables was investigated using receiver operating characteristics (ROCs) and the area under the curve (AUC). All statistical analyses were performed using the 28th version of the SPSS statistical package.

## 3 Results

A total of 135 adult patients with severe asthma managed in QMH were included. The mean age was 57.3 ± 13.9 years. There were more females (64.4%) and never‐smokers (74.8%). The mean pre‐bronchodilator FEV_1_ was 2.12 ± 0.72 L, 93.3 ± 25.7% predicted. The mean FEV_1_/FVC ratio was 66.8 ± 14.5%. The mean bronchodilator reversibility was 66 ± 134 mL and 3.5 ± 7.0%. The mean FE_NO_ level was 35.2 ± 35.1 parts per billion (ppb). The mean baseline blood eosinophil level was 260 ± 244 cells/μL and 3.59 ± 3.14%. The baseline blood total IgE level was 426 ± 1337 IU/mL. The results are summarized in Table 1.

**TABLE 1:** Baseline demographic and clinical characteristics of patients recruited.

Sex (female) (%)	87 (64.4%)
Age (years), mean ± SD	57.3 ± 13.9
Onset age (years), mean ± SD	29.1 ± 20.7
Ever‐smoker	34 (25.2%)
ACT score, mean ± SD	19.7 ± 4.0
Pre‐bronchodilator FEV_1_ (L), mean ± SD	2.12 ± 0.72
Pre‐bronchodilator FEV_1_ (% predicted), mean ± SD	93.3 ± 25.7
Pre‐bronchodilator FVC (L), mean ± SD	3.19 ± 0.93
Pre‐bronchodilator FVC (% predicted), mean ± SD	110.6 ± 19.3
Pre‐bronchodilator FEV_1_/FVC ratio (%), mean ± SD	66.8 ± 14.5
Post‐bronchodilator FEV_1_ (L), mean ± SD	2.23 ± 0.74
Post‐bronchodilator FEV_1_ (% predicted), mean ± SD	98.6 ± 24.4
Post‐bronchodilator FVC (L), mean ± SD	3.23 ± 0.94
Post‐bronchodilator FVC (% predicted), mean ± SD	112.6 ± 18.6
Post‐bronchodilator FEV_1_/FVC ratio (%), mean ± SD	69.2 ± 13.8
Bronchodilator reversibility (mL), mean ± SD	66 ± 134
Bronchodilator reversibility (%), mean ± SD	3.5 ± 7.0
Co‐morbidities	
Allergic conjunctivitis	45 (33.3%)
Allergic rhinitis	107 (79.3%)
Nasal polyp	22 (16.3%)
Rhinosinusitis	20 (14.8%)
Eczema	60 (44.4%)
Urticaria	40 (29.65%)
Hypertension	31 (23.0%)
Diabetes mellitus	9 (6.7%)
Ischemic heart disease	5 (3.7%)
Obstructive sleep apnoea	11 (8.1%)
Bronchiectasis	2 (1.5%)
Malignancies	7 (5.2%)
Connective tissue disease	0 (0%)
Psychiatric disease	18 (13.3%)
Baseline eosinophil count (× cells/μL), mean ± SD	260 ± 244
Baseline eosinophil percentage (%), mean ± SD	3.59 ± 3.14
Serum IgE level, mean ± SD	425.8 ± 1337.1
Asthma treatment	
Budesonide	37 (27.4%)
Daily dose (microgram), mean ± SD	1262 ± 152
Fluticasone propionate	52 (38.5%)
Daily dose (microgram), mean ± SD	1048 ± 227
Fluticasone furoate	46 (34.1%)
Daily dose (microgram), mean ± SD	200 ± 0
Montelukast	86 (63.7%)
LAMA	53 (39.3%)
Montelukast	86 (63.7%)
Theophylline	7 (5.2%)
Maintenance oral corticosteroid	6 (4.4%)

Abbreviations: ACT, asthma control test; FE_NO_, fractional exhaled nitric oxide; FEV_1_, forced expiratory volume in 1 s; FVC, forced vital capacity; kg, kilogram; m, meter; mL, milliliter; ppb, parts per billion; SD, standard deviation; μL, microliter.

### 3.1 Correlation of FE_NO_ With Spirometry Values

**FIGURE 1:**
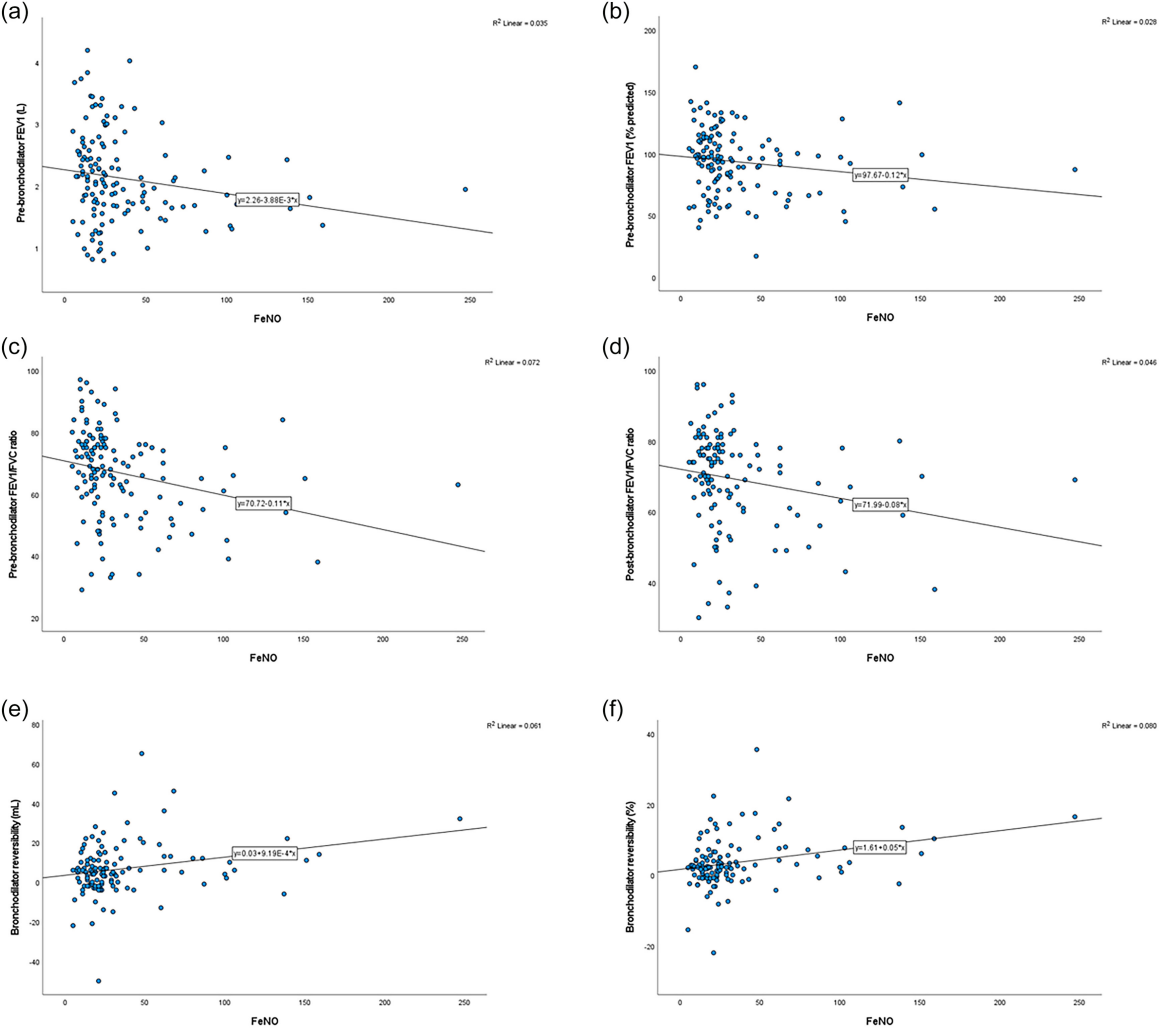
Scatter plots of (a) the correlation between FE_NO_ and pre‐bronchodilator FEV1 (L), (b) the correlation between Fe_NO_ and pre‐bronchodilator FEV_1_ (% predicted), (c) the correlation between Fe_NO_ and pre‐bronchodilator FEV_1_/FVC ratio (*r* = −0.18, *p* = 0.0278), (d) the correlation between Fe_NO_ and post‐bronchodilator FEV_1_/FVC ratio, (e) the correlation between Fe_NO_ and bronchodilator reversibility (mL), and (f) the correlation between Fe_NO_ and bronchodilator reversibility (%).

FE_NO_ was negatively correlated with pre‐bronchodilator FEV_1_ (L) (*r* = −0.188, *p* = 0.029) (Figure 1a), pre‐bronchodilator FEV1 (% predicted) (*r* = −0.169, *p* = 0.050) (Figure 1b), pre‐bronchodilator FEV_1_/FVC ratio (*r* = −0.269, *p* = 0.002) (Figure 1c), and post‐bronchodilator FEV1/FVC ratio (*r* = −0.215, *p* = 0.018) (Figure 1d). FE_NO_ was positively correlated with bronchodilator reversibility (mL) (*r* = 0.248, *p* = 0.006) (Figure 1e) and bronchodilator reversibility (%) (*r* = 0.823, *p* = 0.002) (Figure 1f).

### 3.2 Correlation of FE_NO_ With Blood Eosinophil Level

**FIGURE 2:**
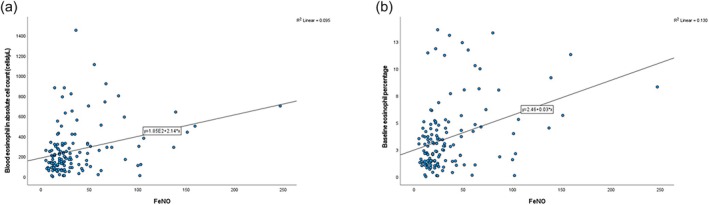
Scatter plots of (a) the correlation between FE_NO_ and blood eosinophil level by absolute cell count and (b) the correlation between FE_NO_ and blood eosinophil level by percentage.

**FIGURE 3:**
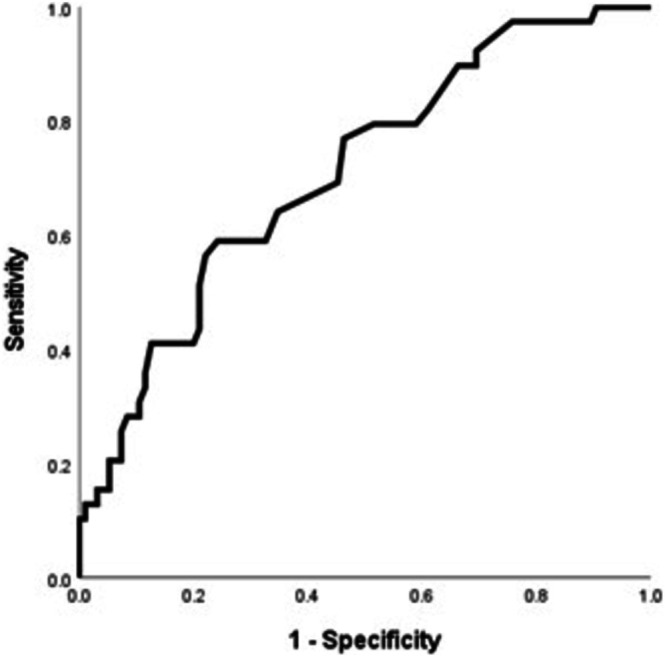
The receiver‐operating characteristic curve analysis of sensitivity and specificity of FE_NO_ for identification of blood eosinophil ≥ 300 cells/μL.

FE_NO_ was positively correlated with baseline blood eosinophil level by absolute cell count (*r* = 0.308, *p* < 0.001) (Figure 2a) and by percentage (*r* = 0.361, *p* < 0.001) (Figure 2b). The ROC curve analysis of the sensitivity and specificity of the FE_NO_ for the identification of blood eosinophil count ≥ 300 cell/mm^3^ at a cutoff point of 24.5 ppb had a sensitivity of 64.1%, specificity of 65.7%, and AUC of 70.9% (95% confidence interval = 0.614–0.804, *p* < 0.0001) (Figure 3).

### 3.3 Correlation of FE_NO_ With Blood Total IgE Level

FE_NO_ was not significantly correlated with baseline blood total IgE level (*r* = −0.087, *p* = 0.315).

### 3.4 Correlation of FE_NO_ With ACT Score

FE_NO_ was not significantly correlated with ACT score (*r* = −0.060, *p* = 0.489).

#### 3.4.1 Subgroup Analysis

In the subgroup with T2‐high asthma, which consisted of 100 patients, FE_NO_ was negatively correlated with the pre‐bronchodilator FEV_1_/FVC ratio (*r* = −0.237, *p* = 0.018). FE_NO_ was positively correlated with blood eosinophil level by absolute cell count (*r* = 0.319, *p* = 0.001) and by percentage (*r* = 0.382, *p* < 0.001), bronchodilator reversibility (mL) (*r* = 0.271, *p* = 0.010), and bronchodilator reversibility (%) (*r* = 0.299, *p* = 0.004).

#### 3.4.2 Sensitivity Analysis

Sensitivity analysis was performed among never smokers. Among the 101 never smokers, FE_NO_ was negatively correlated with pre‐bronchodilator FEV_1_/FVC ratio (*r* = −0.218, *p* = 0.028). FE_NO_ was positively correlated with blood eosinophil level by absolute cell count (*r* = 0.300, *p* = 0.002) and by percentage (*r* = 0.33, *p* = 0.001), bronchodilator reversibility (mL) (*r* = 0.237, *p* = 0.024), and bronchodilator reversibility (%) (*r* = 0.259, *p* = 0.014).

## 4 Discussion

In our study, we observed that FE_NO_ had a possible association between various spirometry markers as well as blood eosinophil count and percentage among adult patients with severe asthma on optimized inhaled treatment. Though the correlation is weak, they reached statistical significance. Our findings suggested that higher prevailing FE_NO_ levels, despite the use of substantial anti‐asthma treatment, might be associated with poorer lung function by FEV_1_, more significant airflow obstruction with a lower FEV_1_/FVC ratio, and more bronchodilator reversibility. All of these pointed to worse asthma control over time and higher prevailing airway hyper‐responsiveness. While FE_NO_ is a marker of airway eosinophilic inflammation, our study showed that it correlated with blood eosinophil count and percentage, which are markers of systemic eosinophilic inflammation with controversial findings reported before.

Despite the potential role of FE_NO_ as suggested by the ATS guidelines to determine glucocorticoid responsiveness as well as guiding asthma treatment [26, 27], criticisms on FE_NO_ in asthma management do exist. Different guidelines and recommendations have different opinions regarding the use of FE_NO_, with GINA suggested to be used for phenotyping [14], while ERS considered it to be a diagnostic tool as well [17]. Systematic reviews also found mixed evidence regarding the use of FE_NO_ to guide asthma therapy [28, 29, 30, 31].

There were pediatric studies to suggest that FE_NO_ was significantly correlated with asthma symptoms within the past 2 weeks, dyspnea score, daily use of rescue medications, and reversibility of airflow obstruction [32], peripheral blood eosinophils, IgE, serum eosinophil cationic protein level, bronchodilator response, and FEV_1_ PC20 methacholine [33]; both studies could not demonstrate significant correlation with FEV_1_ in both liters and percent predicted.

There were studies in the adult population to suggest FE_NO_ correlated with ACT scores [10, 11, 13, 14], blood eosinophil level [12], blood total IgE level [9], positive skin‐prick test, the presence of allergic rhinitis, the presence of allergic conjunctivitis, history of emergency room visits, and spirometry indicators of airway obstruction [13]. At the same time, there were also studies criticizing the role of FE_NO_ in steroid‐treated patients, as the correlation of FE_NO_ with the symptoms, blood, and sputum eosinophil level in steroid‐treated patients was not as good as in steroid‐naïve patients [34]. However, in the latest GINA recommendation, ICS is recommended for treatment across all stages of asthma, and the usefulness of FE_NO_ only among steroid‐naïve patients with mild asthma may not have clinical relevance. Also, FE_NO_ value was not shown to be correlated with lung function parameters in other studies among patients in clinical remission, pediatric and adolescent patients, as well as patients treated with ICSs [12, 15, 35]. The heterogeneous population included was the major limitation from the above studies, leading to controversial results reported.

In a recently published retrospective study, it suggested that blood eosinophil count, IgE, and spirometric values were correlated with the severity of the eosinophilic airway inflammation as measured by Fe_NO_ [36]. However, as pointed out by the author, the limitation of the study is using the retrospective analysis of data from a single pulmonary function laboratory with some of the variables measured on separate occasions.

The controversies about the correlation of Fe_NO_ with clinical, spirometric, and blood biomarkers results could be related to a heterogeneous population being included. Our study design has several strengths that may help to address the controversies in existing literature. First of all, all patients have severe asthma as per International ERS/ATS guidelines on definition, evaluation, and treatment of severe asthma definition. As all the patients were on high dose ICS for severe asthma, the heterogeneity from treatment perspective, especially ICS usage, is no longer present. Their self‐reported adherence has also been evaluated in an asthma patient education program. This could resolve the criticism from previous studies on the role of Fe_NO_ among ICS‐treated asthma patients [34]. Secondly, as a prospective study, all study assessments were done in a clinically stable state and collected within a short interval. This study design has the advantage over other retrospective studies in which the timing of the assessments was not standardized. This can overcome the issues such as variation in ACT score, Fe_NO,_ blood eosinophil, and IgE level from time to time, especially among patients with severe asthma [37, 38]), which has a more fluctuating mild asthma [38, 39].

There are a few limitations in our study. First, this study involved only a single center. However, being a tertiary medical center, the respiratory unit of QMH received referrals from all other health care facilities across the territory. Patients diagnosed with asthma were managed in a designated asthma clinic in our center. FE_NO_ value could be affected by various factors, such as the use of corticosteroid in both inhalation and systemic routes, certain food intake, smoking status, and exercise. To limit the variation of external factors affecting FE_NO_ measurement, FE_NO_ was measured in this study at a clinically stable state away from any exacerbation, systemic steroid use, and change in maintenance treatment (including change in ICS doses), while residual impact from other factors may not be completely avoidable. As a prospective cross‐sectional study, the serial changes in FE_NO_ and blood eosinophil have not been monitored. All the patients included in the study were at stable state on stable medication regimes and we do not expect a major variation of these values to affect the results. The correlation we observed is weak and this could be related to the small sample size which includes asthma patients with different clinical phenotypes. Having a larger scale study with large sample size in each phenotype would be valuable to reexamine the same phenomenon.

## 5 Conclusion

In adult patients with severe asthma, FE_NO_ might have a negative correlation with the FEV_1_, FEV_1_/FVC ratio, and a positive correlation with bronchodilator reversibility, as well as with blood eosinophil levels.

## Author Contributions

The abstract of this manuscript has been presented in the 27th Congress of the Asian Pacific Society of Respirology (APSR 2023) as a poster presentation.

## Ethics Statement

The study was approved by the Institutional Review Board of the University of Hong Kong and Hospital Authority Hong Kong West Cluster. Patient informed consent has been obtained.

## Consent

The authors have nothing to report.

## Conflicts of Interest

The authors declare no conflicts of interest.

## Data Availability

Research data are not shared.
